# Bovine rhinitis B virus is highly prevalent in acute bovine respiratory disease and causes upper respiratory tract infection in calves

**DOI:** 10.1099/jgv.0.001714

**Published:** 2022-02-07

**Authors:** Shaurav Bhattarai, Chun-Ming Lin, Gun Temeeyasen, Rachel Palinski, Feng Li, Radhey S. Kaushik, Ben M. Hause

**Affiliations:** ^1^​ Department of Biology and Microbiology, South Dakota State University, Brookings, SD, USA; ^2^​ Department of Veterinary and Biomedical Sciences, South Dakota State University, Brookings, SD, USA; ^3^​ Animal Disease Research and Diagnostic Laboratory, South Dakota State University, Brookings, SD, USA; ^4^​ Kansas State Veterinary Diagnostic Laboratory and Department of Diagnostic Medicine/Pathobiology, Kansas State University, Manhattan, KS, USA; ^5^​ Department of Veterinary Science, University of Kentucky, Lexington, KY, USA

**Keywords:** bovine rhinitis virus, bovine respiratory disease, picornavirus

## Abstract

Bovine respiratory disease (BRD) is the most significant cause of cattle morbidity and mortality worldwide. This multifactorial disease has a complex aetiology. Dogma posits a primary viral infection followed by secondary bacterial pneumonia. Bovine rhinitis B virus (BRBV) is an established aetiological agent of BRD, but little is known regarding its pathogenesis. Here, a BRD PCR panel identified 18/153 (11.8 %) lung samples and 20/49 (40.8 %) nasal swabs collected from cattle with respiratory signs as positive for BRBV, which was the most prevalent virus in nasal swabs. Primary bovine tracheal epithelial cells were used to isolate BRBV that was phylogenetically related to contemporary sequences from the USA and Mexico and genetically divergent from the previous sole BRBV isolate. To investigate virus pathogenesis, 1-week-old colostrum-deprived dairy calves were inoculated intranasally with 7.0 log_10_ TCID_50_ BRBV. Virus was isolated from nasal swabs, nasal turbinates, trachea and the brain of the challenged animals. Neutralizing antibodies were detected beginning 7 days post-inoculation and peaked at day 14. *In situ* hybridization (ISH) localized BRBV infection in the upper respiratory ciliated epithelial and goblet cells, occasionally associated with small defects of the superficial cilia lining. Sporadically, pinpoint ISH signals were also detected in cells resembling glial cells in the cerebrum in one calf. Together, these results demonstrate the BRBV infection is highly prevalent in acute BRD samples and while the pathogenicity of BRBV is minimal with infection largely limited to the upper respiratory tract, further research is needed to elucidate a possible initiatory role in BRD.

## Introduction

The species Bovine rhinitis B virus (BRBV) is a non-enveloped, single-stranded, positive-sense RNA virus belonging to the genus *Aphthovirus* of the family *Picornaviridae* [1]. The genus *Aphthovirus* also includes equine rhinitis A virus (ERAV), bovine rhinitis A virus (BRAV) and foot and mouth disease virus (FMDV), the last being one of the most significant livestock viruses worldwide to which BRBV is closely related. Bovine rhinitis A virus consists of two serotypes, BRAV1 and BRAV2, while only a single serotype of BRBV has been described [2, 3]. The increasing use of next generation sequencing has revealed considerable genetic diversity present in BRBV [4], the impact of which on antigenicity is unknown.

Formerly bovine rhinovirus, BRAV was first isolated in Germany in 1962 and has been reported from the USA, England, Japan, Italy, Sudan and Mexico [3, 5–12]. Originally classified as a serotype of bovine rhinovirus, the sole isolate for BRBV originated from a calf with respiratory disease in England in 1964 [13]. Few pathogenesis studies for bovine rhinitis viruses have been conducted, largely in the 1960s and 1970s, owing in part to the difficulty in propagating virus in cell culture. Experimental inoculation of colostrum-deprived calves with BRAV1 strain RS 3 x resulted in the development of focal rhinitis, with virus isolated from nasal swabs up to 14 days post-inoculation [14]. Neutralizing antibody titres were detected 14 days after inoculation that persisted for at least 5 months. Gnotobiotic calves inoculated via intratracheal and intranasal routes with BRBV strain EC-11 developed asymptomatic interstitial pneumonia with severe focal rhinitis [15]. In a separate study, pretreatment of calves with bovine fibroblast IFN resulted in lower shedding of bovine rhinitis virus in nasal secretions compared to non-treated controls [16]. Generation of cell-mediated immunity, peaking from days 4 to 12 prior to the neutralizing antibody response, has also been observed following experimental inoculation of BRAV1 [17].

Bovine respiratory disease (BRD) is the most economically significant disease of the cattle industry, accounting for more than $1 billion in annual losses in the USA alone [18]. In the USA, BRD accounts for 70–80 % morbidity and 40–50 % mortality in feedlots [19]. The aetiology of BRD includes interactions of pathogens, environmental factors, management factors, physiological factors and stress [20]. Pathogenesis of BRD is thought to initiate with viral infection in the respiratory mucosa followed by secondary bacterial infection by commensal bacteria, leading to pneumonia [21]. In the upper respiratory tract, BRD histopathology includes rhinitis and tracheitis [22]. Commercially available vaccines for BRD include both inactivated and attenuated bacteria and viruses but not BRBV. Commonly included bacteria in these vaccines are *Mannheimia haemolytica, Histophilus somni* and *Pasteurella multicoda,* and viruses included are bovine respiratory syncytial virus (BRSV), bovine herpesvirus 1 (BHV1), bovine viral diarrhoea virus (BVDV) and parainfluenza virus 3 (PI3) [22]. Despite widespread use of vaccines and antibiotics, the incidence of BRD has been increasing [19, 23].

Despite being established aetiological agents of BRD, BRAV and BRBV have been neglected for decades; however, more widespread use of NGS in recent years suggests that bovine rhinitis viruses play a clinical role in BRD [24, 25]. A viral metagenomic study of nasal swabs collected from feedlot cattle with BRD along with healthy pen mates in the USA and Mexico found BRAV and BRBV were the most prevalent viral pathogens, with detection in 52.7 and 23.7 % of nasal swabs, respectively [3]. A similar study conducted in Canadian feedlots found a high prevalence of BRBV (21.9%) in deep nasal swabs collected from calves on arrival at feedlots [26]. Another feedlot study performed in Canada found that detection of both BRAV and BRBV, in addition to influenza D virus (IDV), bovine coronavirus (BCV) and BRSV, in nasal swabs and tracheal washes were significantly associated with BRD [25]. Metagenomic sequencing of dairy calves with BRD and site-matched healthy controls at a large California calf ranch found that BRAV, bovine adenovirus 3 and IDV were all significantly associated with BRD [24]. The high prevalence of bovine rhinitis viruses in bovine calves and feedlot cattle, variably associated with BRD, highlights the need to revisit the role of bovine rhinitis viruses in BRD pathogenesis and epidemiology.

In this study, we isolated BRBV from a nasal swab collected from a calf with clinical respiratory disease. Following intranasal inoculation of naïve calves, the virus was isolated up to 8 days post-inoculation (dpi) from nasal swabs, neutralizing antibody was detected after 7 dpi, and *in situ* hybridization revealed BRBV infection in the epithelial cells of nasal mucosa, trachea and brain. We also studied the prevalence of common bovine respiratory pathogens in nasal swab and lung samples sent for BRD diagnosis and found that BRBV was the most common virus identified in nasal swabs from symptomatic cattle

## Methods

### Prevalence of BRDC pathogens

The prevalence of common bovine respiratory pathogens was studied in nasal swab and lung samples submitted for BRD PCR panel testing at the Kansas State Veterinary Diagnostic Laboratory as part of routine herd management and veterinary care. Samples were collected from farms in Kansas between 1 October 2020 and 27 January 2021. Quantitative reverse transcriptase PCR (qRT-PCR) was used to detect viral and bacterial pathogens. Bacterial pathogens included in the PCR panel were *Mycoplasma bovis, H. somni, Mannheimia haemolytica, Bibersteinia trehalosi* and *P. multicoda*. Viral pathogens included in the panel were BVDV, BHV1, BRSV, BCV and IDV. Samples were additionally screened for BRBV [27]. All the samples were collected from the cattle exhibiting clinical respiratory disease.

### Cell culture and virus isolation

Nasal swabs submitted to the South Dakota State Animal Disease Research and Diagnostic Laboratory for BRD testing were screened for BRBV by quantitative RT-PCR. Virus isolation was attempted on positive samples with Ct values <25 (*n*=22). Insufficient sample quantities prevented virus isolation from samples used for the BRBV prevalence study. Primary bovine tracheal epithelial cells developed in an earlier study were used to isolate BRBV [28]. Cells were grown in DMEM/F-12 media supplemented with 5 % FBS, epidermal growth factor (Corning; cat. no. 354001), insulin-transferrin-selenium (Corning; cat. no. 354350) and 1× antibiotics-antimycotic (Gibco; cat. no. 15240062) at 33 °C with 5 % CO_2_. Confluent cells were inoculated at a volumetric 1 : 1000 dilution of 0.22 µm filtered nasal swabs resuspended in PBS. Infected cells were cultured at 33 °C in 5 % CO_2_ for 5 days. Virus isolation was confirmed by cytopathic effects (CPEs), qRT-PCR and genome sequencing.

### Metagenomic sequencing and phylogenetic analysis

Metagenomic sequencing was performed as previously described [4]. Paired 151 bp reads were assembled *de novo* in CLC Genomics and identified by blastx using the integrated Blast2Go plugin and the GenBank non-redundant protein database.

Metagenomic sequencing was performed on BRD submissions used for the BRBV prevalence study with qRT-PCR Ct values <25 for BRBV (*n*=7) and isolated virus BRBV strain 6900. Complete coding DNA sequences and partial non-coding regions of the genomes were assembled.

The genomes for BRBV strains 6900, B2 and D10 were aligned with all BRBV genomes in Genbank by clustalw as implemented in megax. Phylogenetic analysis was performed by maximum-likelihood analysis using the best-fitting GTR+G+I model of evolution using BRBV genome sequences with tree topology evaluated with 1000 bootstrap replicates. Sites with <80 % coverage were omitted from the analysis.

### BRBV pathogenesis in calves

Male Holstein calves were caught at birth without touching the bedding and immediately transferred to the biosafety level 2 animal facility. The calves did not receive colostrum. Mock and BRBV-challenged calves were housed in separate rooms. Calves were individually penned but had direct contact with other calves in the room. Calves were bottle fed twice daily with human infant formula on arrival until day 3 when they were transitioned to bovine milk replacer. The umbilical cord area was treated daily with chlorhexidine on days 0–3 and calves were orally given 10 ml of commercial Clostridium C/D antitoxin on days 1–3 in addition to the commercial antibiotic Baytril. Calves were allowed 7 days to acclimate prior to study initiation.

Five calves were used for the study. Three calves (201, 203 and 205) were challenged intranasally with 10 ml of 6.0 log_10_ TCID_50_ ml^–1^ of BRBV strain 6900. Two calves (204 and 206) were mock-inoculated with PBS.

Calves were observed daily and clinical scores were recorded as previously described [29]. Beginning on the day of challenge (day 0), calves were swabbed daily until they were qRT-PCR negative for two consecutive days (qRT-PCR Ct >35). Blood samples were collected on days 0, 3, 5, 7, 10, 14 and 21 for serological analysis. Three calves were killed on day 5, two from the virus-challenged group (calves 201 and 203) and one from the control group (calf 204). The remaining calf from each group was killed 21 dpi. Nasal swab, nasal turbinate, upper and lower trachea, tracheobronchial lymph node, lung, brain, kidney, liver, spleen, bladder, mediastinal lymph node, mesenteric lymph node, heart, small and large intestine, and blood samples were collected at necropsy.

### Serum neutralization assay

Sera were heat-inactivated at 56^ ^°C for 30 min then serially diluted in DMEM/F12 media. Equal volumes of sera were combined with 100 TCID_50_ of virus at 33 °C for 1 h. The serum and virus suspension was used to inoculate primary bovine tracheal epithelial cells, which were observed for 5 days for CPEs. The neutralizing antibody titre was calculated as the reciprocal of the highest antibody dilution capable of neutralizing 100 TCID_50_ of virus.

### qRT-PCR

Viral RNA was isolated using a commercially available kit (QIAamp viral RNA kit, ref 52906). qRT-PCR was performed using a one-step qRT-PCR kit (Taqman Fast virus 1-step master mix, ref. 4444432; Applied Biosystems) following the manufacturer’s protocol. The Taqman assay used in the study was previously reported [28]. For amplification, a 7500 Fast Real time PCR system (7500 system v2.3; Applied Biosystems) was used with the following thermal cycling conditions: 50 °C for 5 min, 95 °C for 20 s then 40 cycles of 95 °C for 15 s and 60 °C for 60 s.

A DNA fragment encompassing the region targeted by the qRT-PCR assay flanked by a 5′-T7 promoter sequence was synthesized and used to generate a synthetic RNA transcript using a MAXIscript kit following the manufacturer’s instructions. The concentration of the resulting RNA was determined using a Qubit RNA kit following digestion with DNase I. Serial 10-fold dilutions of synthetic RNA were used to generate a standard curve to correlate RNA copies to the qRT-PCR Ct value (Fig. S1, available in the online version of this article).

### Histopathology

Tissues were fixed overnight in 10 % neutral buffered formalin. Tissues were routinely processed at the South Dakota State University Animal Disease Research and Diagnostic Laboratory. Haematoxylin and eosin (H&E)-stained slides were used for histopathological examination.

### *In situ* hybridization of BRBV

*In situ* hybridization was performed on formalin-fixed paraffin-embedded (FFPE) tissue using a commercially available kit (RNAscope 2.5 HD detection reagent-RED, cat. no. 322360-USM). Slides were deparaffinized, re-hydrated and blocked with hydrogen peroxide. Following antigen retrieval, sections were hybridized with the probe designed against the 3D polymerase gene region of the BRBV genome. The presence of BRBV in the tissue section was visualized using the fast red substrate. *In situ* hybridization was performed in duplicate on four sections of nasal turbinates, three sections of trachea, six sections of lung and two sections of cerebrum.

### Virus titration

Tissue homogenates were prepared from the nasal mucosa, upper and lower trachea, lungs, mediastinal lymph node, tonsil and brain collected on day 5. Tissue samples were homogenized in Hanks’ balanced salt solution, pH 7.4, centrifuged at 12 000 ***g*** for 5 min, filtered through a 0.22 µm filter and stored at −80 °C. For titration, primary bovine tracheal epithelial cells were seeded in 96-well plates and upon 70 % confluency 10-fold serial dilutions of the tissue homogenates were used to inoculate cells. Cells were cultured at 33 °C and 5 % CO_2_ for 5 days. Cells were observed for visible CPEs. Virus titre was determined using the Reed and Muench method of determining 50 % endpoints [30].

## Results

### BRBV is highly prevalent in nasal swabs from clinical BRD diagnostic submissions

**Fig. 1.**
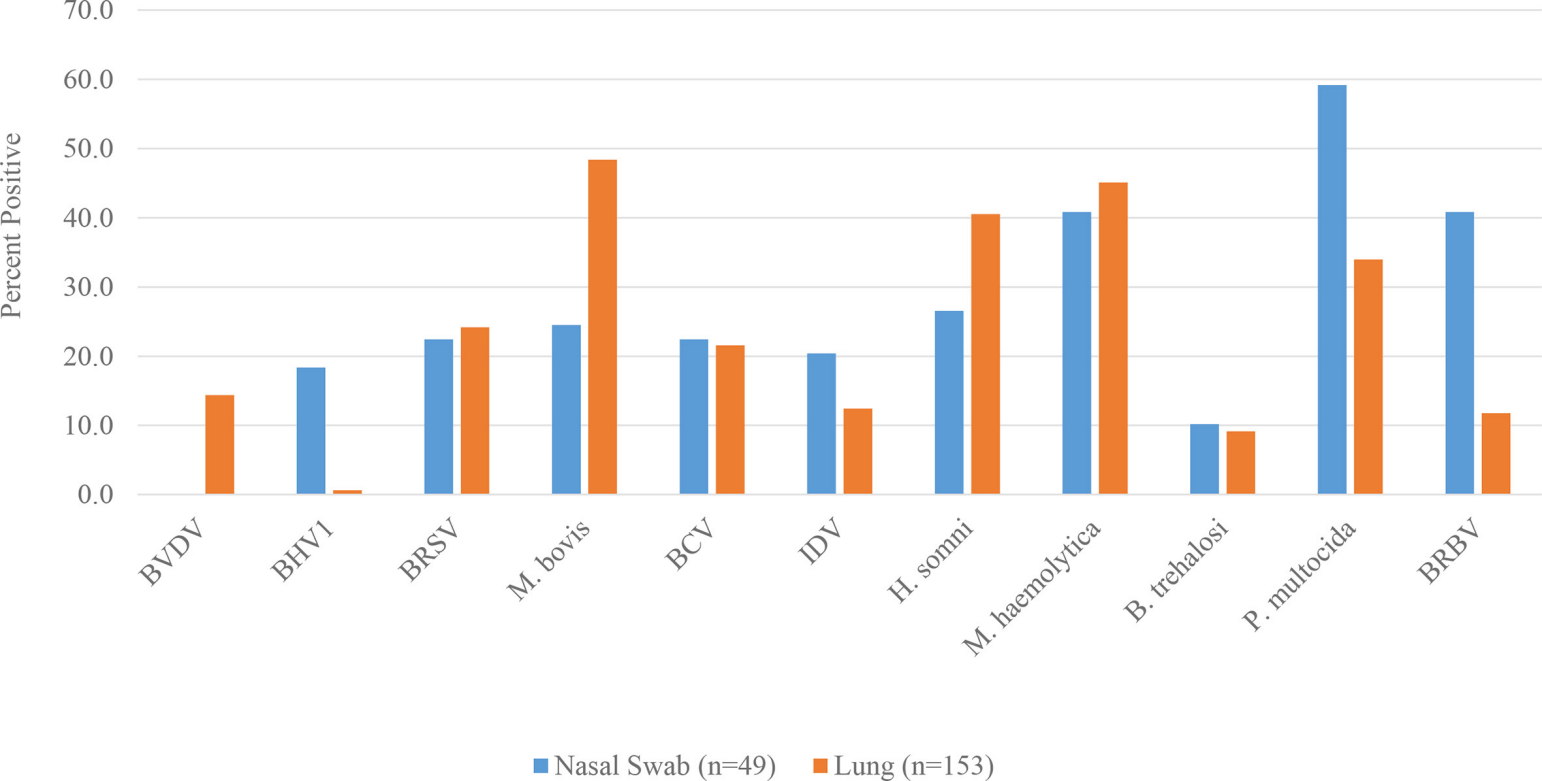
Percentage of nasal swabs and lung tissues submitted for bovine respiratory disease panel PCR that are positive for BRD-associated pathogens. Bovine viral diarrhoea virus, BVDV; bovine herpesvirus 1, BHV1; bovine respiratory syncytial virus, BRSV; *Mycoplasma bovis *; bovine coronavirus, BCV; influenza D virus, IDV; *Histophilus somni *; *Mannheimia haemolytica *; *Bibersteinia trehalosi *; *Pasteurella multocida *; bovine rhinitis B virus, BRBV.

A collection of 49 nasal swabs and 153 lung samples submitted for BRD diagnostic testing were analysed for bacteria and viruses associated with BRD by qRT-PCR. Bovine rhinitis virus was detected in 20 of 49 (40.8 %) nasal swabs but only in 18 of 153 (11.8 %) lung samples (Fig. 1). BRBV was detected nearly twice as frequently as the next most prevalent virus, BCV, detected in 22.4 % of nasal swabs. In contrast, all viruses except BHV1 were more prevalent than BRBV in lung samples. Interestingly, 6 % (3/49) of the nasal swab samples and 0.7 % of the lung samples (1/153) were only positive for BRBV.

Metagenomic sequencing was performed on seven of the BRBV-positive samples that had the lowest Ct values for BRBV. Complete BRBV genomes were assembled for strains D2 and B10. These sequences were submitted to GenBank under accessions OL410605 and OL410606.

### Isolation of BRBV on primary bovine tracheal epithelial cells *in vitro*

Nasal swabs collected from cattle with clinical BRD and submitted for diagnostic testing were used for virus isolation. BRBV strain 6900 was isolated from a nasal swab collected from a calf in California with acute respiratory disease on primary bovine tracheal epithelial cells. Five days post-inoculation, a CPE was evident and virus growth was confirmed by qRT-PCR (Ct <20), and NGS was used to determine the viral genome sequence and to confirm the absence of any exogenous organisms. All of the sequencing reads, excluding those derived from the host cell *Bos taurus,* mapped to BRBV and we were able to obtain a near complete BRBV genome sequence of 7466 nt. Strain 6900 was submitted to GenBank as accession MZ574106. A blastn search of the NCBI nucleotide database identified a maximum of 89.5 % identity to a BRBV genome from Mexico (GenBank accession KU159357). The isolated BRBV strain 6900 was 79.7 % identical to the reference BRBV strain EC-11.

### Phylogenetic analysis identified co-circulation of diverse BRBV strains in cattle

**Fig. 2.**
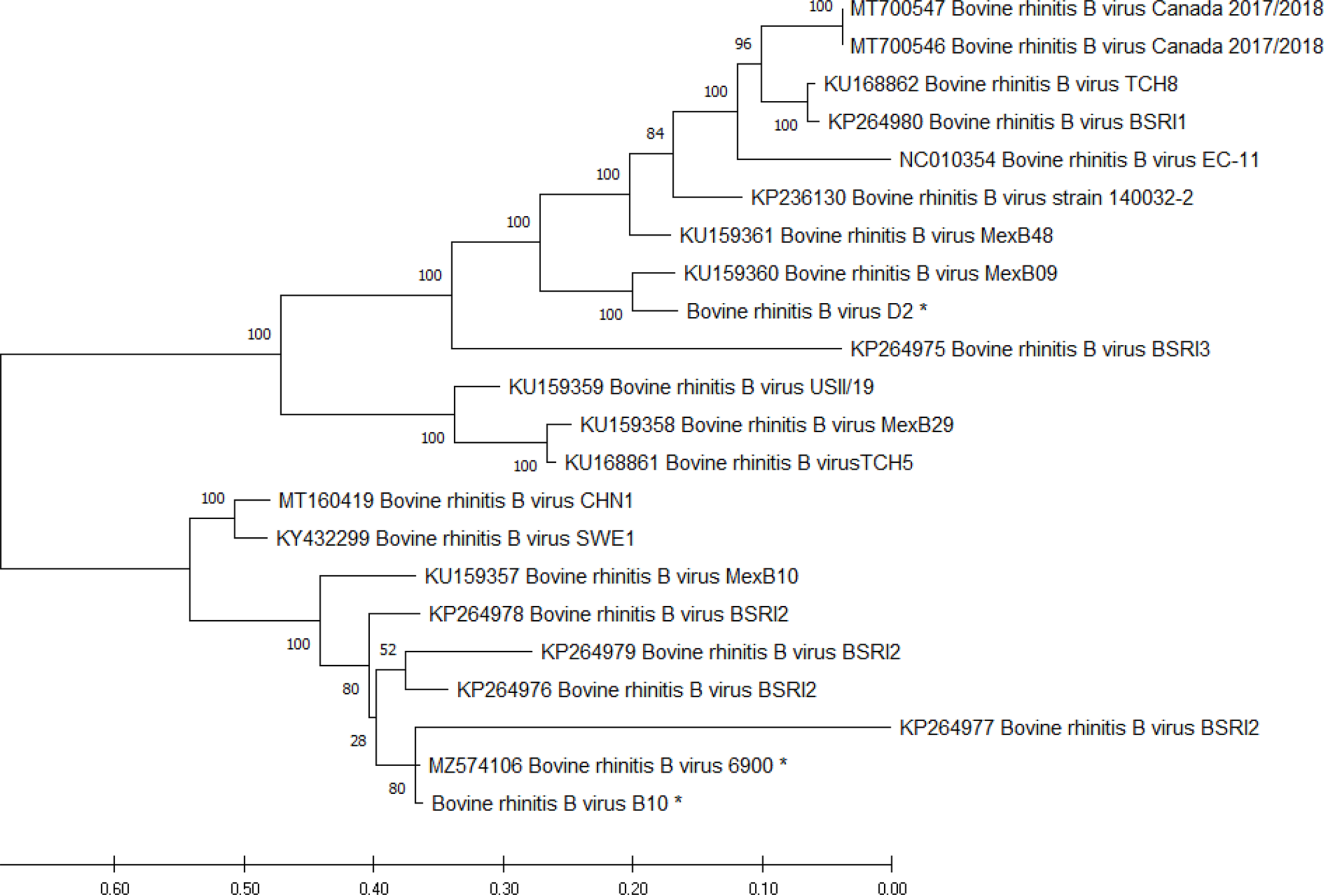
Phylogenetic analysis of the complete genomes of BRBV strains 6900, D2 and B10 and 19 reference genomes. Nucleotide sequences were aligned by clustalw with the phylogenetic tree inferred by maximum-likelihood analysis using the GTR+G+I model of evolution using 1000 bootstrap replicates. BRBV genome sequences determined here are indicated by an asterisk. Scale bar matches that determined in the phylogenetic analysis software.

To explore the genetic diversity and evolutionary relationships of BRBV strains, phylogenetic analysis was performed with all publicly available BRBV genomes (*n*=19) and the three genomes determined here. Strains 6900 and B10 clustered with four genomes recovered from calves with BRD from a large calf ranch in California, designated BSRI2 (Fig. 2). BRBV sequences formed two distinct, well-supported clades, with strain 6900 included in a lineage with contemporary genomes from China, Sweden and Mexico. BRBV strain D2, the BRBV reference strain EC-11, along with diverse viruses from the USA, Canada and Mexico, were found in the other major clade. There was approximately 15–20 % intraclade and 25 % interclade divergence. The identification of BRBV strains B10 and D2 from geographically and temporally closely related farms suggests concurrent circulation of both genotypes of BRBV and demonstrates that considerable genetic diversity exists for contemporary BRBV.

### Inoculation of calves with BRBV resulted in minimal clinical signs

Three 7-day-old calves were inoculated intranasally with 10^7^ TCID_50_ of BRBV strain 6900 and observed daily for clinical signs. The only clinical sign noted throughout the experiment was calf 201 had a fever (>39.40 C) on day 2 post-challenge which resolved by day 3. No other clinical signs were observed.

### BRBV infection is primarily associated with the upper respiratory tract

**Fig. 3.**
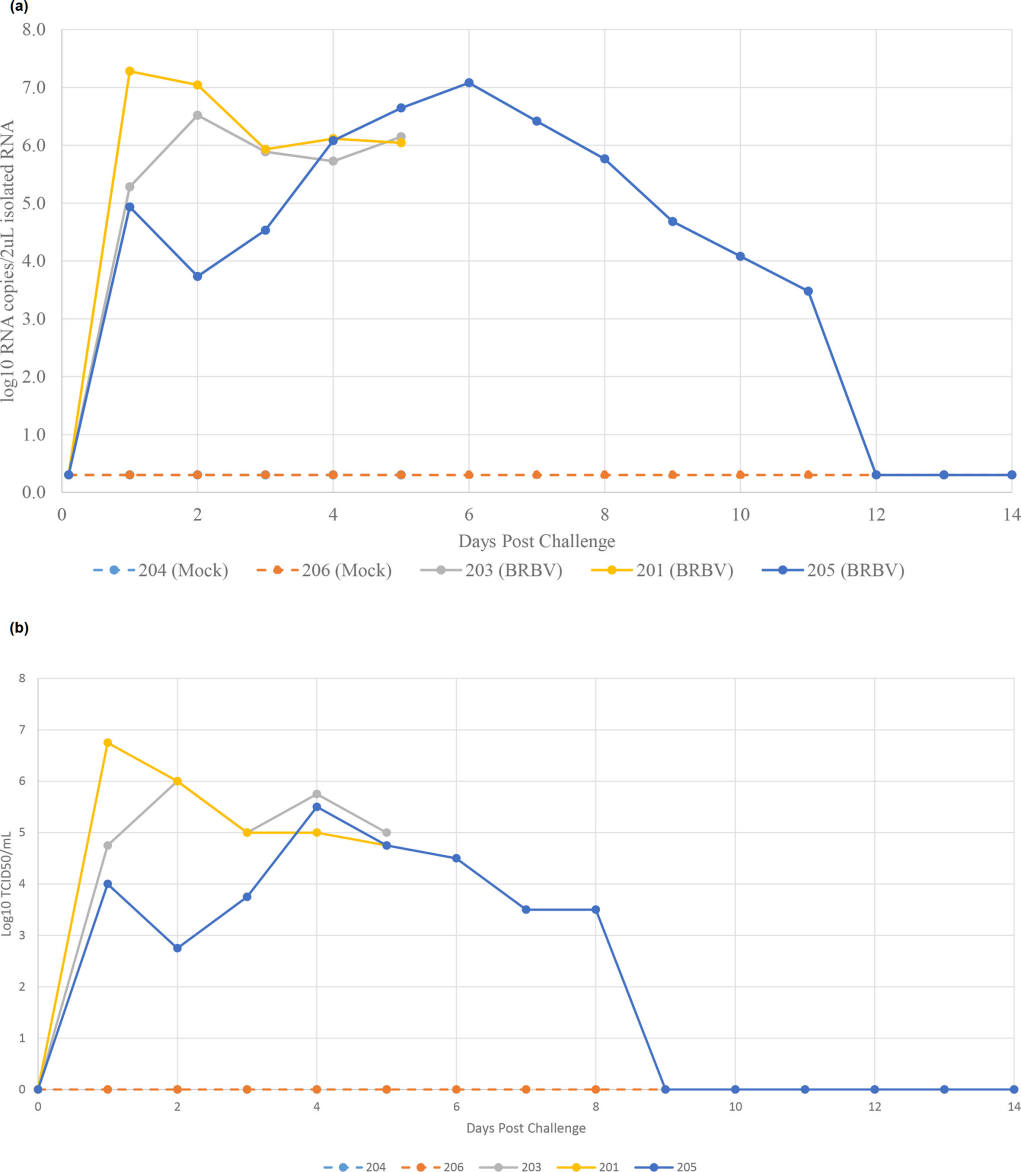
BRBV shedding in nasal swabs detected by (a) qRT-PCR and (b) titration on primary bovine tracheal epithelial cells. Calves 201, 203 and 205 were intranasally inoculated with BRBV on day 0 while calves 204 and 206 were mock inoculated. Calves 201, 203 and 204 were killed on day 5.

Nasal swabs were analysed by qRT-PCR. All calves were negative for BRBV on the day of challenge. BRBV RNA was detected in nasal swabs from day 1 to 11 for BRBV-challenged calves (Fig. 3a). Mock challenged calves remained negative throughout the study. Titration of nasal swabs gave similar results to qRT-PCR, with rapid onset of BRBV shedding detected beginning on day 1 and lasting until day 8 (Fig. 3b).

Tissues collected at 5 dpi from two BRBV and one mock-challenged calves were analysed by qRT-PCR. Nasal turbinates and trachea samples from both BRBV inoculated calves were positive (Table 1). For calf 203, tonsil, mediastinal lymph node, brain, right accessory lung lobe, bladder, small intestine, large intestine and heart were also positive for BRBV by qRT-PCR (Table 1). By titration, only the nasal turbinate samples were positive for both calves. The upper trachea and brain tissue homogenates for calf 203 were additionally positive for BRBV by titration. Sera samples on 0, 3, 5, 7, 10 and 14 dpi were all negative for BRBV by qRT-PCR (Table 1).

**Table 1.** Detection of BRBV in tissues from calves killed 5 days post-inoculation −, Negative; +, positive; nt, not tested; LN, lymph node.

	201 (BRBV)			203 (BRBV)			204 (Mock)		
Tissue	RT-PCR*	TCID_50_†	ISH	RT-PCR	TCID_50_	ISH	RT-PCR	TCID_50_	ISH
Nasal turbinate	5.7	4.0	+	5.2	3.75	+	−	−	−
Tonsil	−	−	nt	3.7	−	nt	−	−	nt
Upper trachea	3.6	−	+	4.3	2.5	+	−	−	−
Lower trachea	2.9	−	−	2.5	−	−	−	−	−
Tracheobronchial LN	−	nt	nt	−	nt	nt	−	nt	nt
Mediastinal LN	−	−	nt	3.6	−	nt	−	−	nt
Mesenteric LN	−	nt	nt	−	nt	nt	−	nt	nt
Brain	−	−	−	3.4	1.75	+	−	−	−
Right cranial lung	−	nt	−	−	nt	−	−	nt	−
Right caudal lung	−	nt	−	−	nt	−	−	nt	−
Right middle lung	−	nt	−	−	nt	−	−	nt	−
Right accessory lung	−	nt	−	2.6	nt	−	−	nt	−
Left cranial lung	−	nt	−	−	nt	−	−	nt	−
Left caudal lung	−	nt	−	−	nt	−	−	nt	−
Liver	−	nt	nt	−	nt	nt	−	nt	nt
Spleen	−	nt	nt	−	nt	nt	−	nt	nt
Bladder	−	nt	nt	2.5	nt	nt	−	nt	nt
Small intestine	−	nt	nt	3.7	nt	nt	−	nt	nt
Left kidney	−	nt	nt	−	nt	nt	−	nt	nt
Right kidney	−	nt	nt	−	nt	nt	−	nt	nt
Large intestine	−	nt	nt	2.7	nt	nt	−	nt	nt
Heart	−	nt	nt	3.0	nt	nt	−	nt	nt
Serum	−	nt	nt	−	nt	nt	−	nt	nt

*log_10_ RNA copies per 2 µl isolated RNA.

†log_10_ TCID_50_ ml^−1^.

### Metagenomic sequencing of nasal swabs identified both BRBV and BCV infection of calves

Metagenomic sequencing of pooled nasal swabs collected on the day of challenge unexpectedly identified BCV. Despite transfer of calves to BSL2 isolation immediately after birth, the calves were naturally infected with BCV, presumably due to exposure to BCV-contaminated faecal material during birth. To confirm this finding, qRT-PCR for BCV was performed on individual nasal swabs. All calves were positive with Ct values of 18.1–28.2 on day 0. Likewise, nasal swabs from all calves were positive for BCV on day 1 post-challenge. By day 5, only calves 206 and 203 remained positive for BCV. Complete genomes with >99 % identify to BRBV strain 6900 were assembled from nasal swabs from all BRBV-challenged calves on days 1 and 5 post-inoculation. Apart from BCV, no other pathogens were identified.

### BRBV infection induces a rapid neutralizing antibody response

Both mock-challenged calf 206 and BRBV-challenged calf 205 were negative for neutralizing antibodies at days 0 and 5 post-challenge. Calf 206 remained seronegative to day 21. In contrast, calf 205 had measurable serum neutralizing titres to BRBV strain 6900 of 20, 60, 160 and 80 on days 7, 10, 14 and 21, respectively.

### Minimal histological changes are associated with BRBV infection

Examination of routine H&E-stained slides showed no obvious microscopic lesions in tissue from both BRBV- and mock-inoculated calves. There were small numbers of resident lymphocytes, plasma cells and histiocytes in the mucosa of the upper respiratory tract with no differences between groups.

**Fig. 4.**
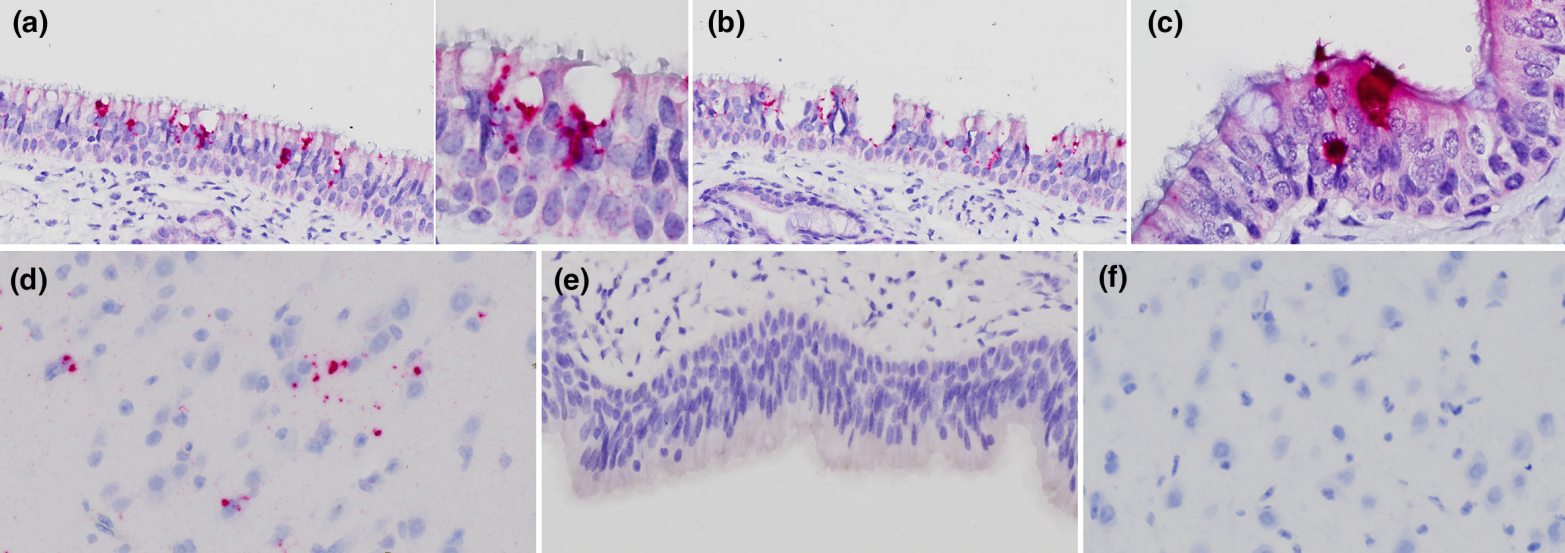
*In situ* hybridization staining of BRBV. Positive BRBV staining is visualized in the nasal mucosa (**a, b**) and trachea (**c**) of the infected calves (calf 201 and calf 203) and cerebrum (**d**) in one calf (calf 203). Hybridized signals were detected as pinpoints or granules beside the nuclei (**a, b**) or diffusely in the cytoplasm (**c**). Occasionally, small defects of the superficial lining cilia were noted (**b**). There was an absence of staining in the nasal mucosa (**e**) and cerebrum (**f**) of the mock-inoculated calf. Tissues were collected 5 DPI. Cells were counterstained with haematoxylin. The images was taken at 400x (**a, b, e**), 600x (**d, f**), and 1000x (**c**) .

In BRBV-inoculated calves, positive *in situ* hybridization (ISH) signals were observed in small clusters and individual epithelial cells, covering approximately 10–25 % of the upper respiratory tract (Fig. 4a, b). The density of ISH-positive cells was subjectively higher in the nasal mucosa than in the trachea. Conversely, no ISH signal was observed in the bronchus and lungs.

At higher magnification, the BRBV genome was hybridized as numerous pinpoints and small dots stippled in the cytoplasm of mucosal ciliated epithelial cells and goblet cells in the nasal turbinates and trachea (Fig. 4a–c). The hybridized signals were perinuclear or adjacent to the secretory vesicles of goblet cells, and occasionally, diffusely obscuring details of the entire cytoplasm. Small defects of the superficial cilia lining were noted (Fig. 4b). Occasionally, a few pinpoint signals of BRBV in cells resembling glial cells and adjacent neuropil in the cerebrum (one calf, 203) were detected (Fig. 4d). Slides of the remaining collected tissues were negative. ISH signal was not detected in tissues collected from mock-inoculated calves (Fig. 4e, f).

## Discussion

Bovine rhinitis viruses are established aetiological agents of BRD, yet little is known on their pathogenesis in BRD. We isolated BRBV from a nasal swab submitted for BRD diagnosis, representing only the second BRBV isolate reported in the half century since initial characterization. Given the difficulty of propagating BRBV in immortalized cell lines, we used primary bovine tracheal epithelial cells established in an earlier study to mimic *in vivo* growth conditions of the virus. Previously isolated BRBV strain EC-11 made use of a bovine tracheal organ culture system, which was later adapted to a bovine kidney cell line [8].

BRBV was detected in 40.8 % of nasal swabs and was the most prevalent virus in nasal swabs. In contrast, BRBV was only detected in 11.1 % of the lung samples where it was one of the least prevalent viruses. More frequent detection of BRBV in nasal swabs than lung tissue is consistent with our pathogenesis study where BRBV replication was largely limited to the upper respiratory tract. Frequent detection of BRBV in the upper respiratory tract of cattle with respiratory disease has been reported. A metagenomic sequencing-based study of nasal swabs from dairy calves in California identified multiple strains of BRBV; 8–10 % of the symptomatic animals but only 2 % of asymptomatic animals were BRBV-positive [24]. A separate metagenomic sequencing study performed in western Canada identified 28 % BRBV-positive cases in BRD symptomatic cattle and 10 % positive in healthy control animals [25]. Interestingly, only three out of 16 symptomatic BRBV-positive animals were positive in both nasal swabs and tracheal washes. Metagenomic sequencing of nasal swabs collected from cattle in US and Mexican feedlots found BRAV and BRBV, together with BCV, as the most frequently identified viruses [3]. A limitation of our study was a lack of nasal swabs from clinically normal animals that would enable possible association of BRBV detection with clinical disease.

This and previous studies used colostrum-deprived or gnotobiotic calves to study BRBV pathogenesis to reduce the interference of maternal antibodies and co-infections [14, 15]. Both our screening of BRD diagnostic submissions for BRBV and other published work has shown that BRBV is endemic in US cattle, with prevalence in acute BRD cases of ~10–40 % [3, 4, 24]. While no serological assays have been developed that are suitable for screening large numbers of sera samples, it is likely that maternal antibodies to BRBV are ubiquitous in calves, necessitating use of colostrum-deprived animals to study virus pathogenesis in the absence of maternal antibody interference.

While epidemiological studies have highlighted the prevalence of BRBV, little information regarding the pathogenicity is available. To our knowledge, there is only one experimental inoculation study, published in 1971 [15]. Three gnotobiotic calves were inoculated with organ-cultured BRBV strain EC-11. A few small dark red areas in the lungs were the only gross changes. The virus was re-isolated from the lungs, tonsils and turbinates. In nasal turbinates and trachea, few foci of epithelial necrosis, enhanced mucus activity and exocytosis of leukocytes into the epithelial and subepithelial tissues were observed. Microatelectasis and interstitial pneumonia were also reported from the BRBV challenge study. In the present study, our observations were generally compatible with those reported earlier. Only minimal histopathological changes were observed. ISH, combined with qRT-PCR and titration, demonstrated that infection and replication of BRBV was mainly in the upper respiratory tract and differentiated the virus-associated changes from non-specific background lesions. Despite detection of BRBV in 11.8 % of lung samples, we did not find any evidence of BRBV infection of lungs in our calf inoculation study.

In the upper respiratory tract, pathogen invasion is prevented by innate immune responses including a mucus barrier and ciliary movement. Viruses that overcome these barriers can infect the upper respiratory tract, possibly predisposing the host to further infection of the lower respiratory tract and co-infection with commensal bacteria [22]. Our study demonstrated BRBV infection in nasal and tracheal mucosal epithelial cells, associated with isolated cell death. In addition to histological lesions, a previous study found BRBV infected tracheal cells showed reduced ciliary activity after 7 days of infection, suggesting changes at the functional level [8]. Our results confirm and expand upon previous studies and demonstrate that BRBV infects the epithelial cells of the upper respiratory tract and may play a role in creating favourable conditions for commensal opportunistic pathogens to invade the mucosal layer, thus initiating BRD pathogenesis.

Picornaviruses such as poliovirus are often associated with nervous system infection [31]. Pathogenesis studies on the closely related FMDV using C57BL/6 and BALB/C mice demonstrated viral replication in the brain [32, 33]. In our study, we isolated BRBV from the brain of one calf killed 5 dpi, corresponding to a positive result by qRT-PCR. Using ISH, BRBV genomes were hybridized as few cytosolic pinpoints near the nucleus of cells resembling glial cells or scattered throughout the neuropil. However, no neurological signs or microscopic lesions were observed. This is the first report of neurotropism for BRBV. However, the clinical relevance of BRBV infection of the brain and underlying pathogenesis needs further study.

Unexpectedly, NGS of pooled day 0 nasal swabs identified BCV infection that was confirmed by qRT-PCR. While all five calves were positive for BCV at day 0, only two remained positive by day 5, suggesting that BCV infection was waning. We speculate that the calves were infected at birth due to BCV-contaminated faecal material present on the cows. Aside from BCV, no other pathogens were identified. Importantly, we failed to detect BCV in the nasal turbinates, trachea, lungs and intestine tissues collected on day 5 by immunohistochemistry (data not shown). Although BCV is a known aetiologic agent of BRD, it is also commonly carried in asymptomatic animals [34–36]. BCV was the second most prevalent virus detected in nasal swabs collected from acute BRD cases screened here. Given the clinical signs and pathological changes in BRBV-inoculated calves are minimal to absent, we believe the contamination of BCV is unlikely to have a significant effect on the results of our study.

Reproduction of significant clinical disease with BRBV is challenging. Clinical disease was not apparent in our pathogenesis study, with histological lesions relatively minor and limited to the upper respiratory tract. The ability of BRBV to serve as an initiator of BRD is unknown but warrants further study. In addition to costs directly attributed to clinical disease, economic losses from subclinical BRD are estimated at around $50 and 16 kg of carcass per animal [37]. Inclusion of bovine rhinitis virus to routine BRD diagnostics and vaccines should be considered. Further studies are needed to establish the significance of BRBV on BRD pathogenesis.

## Supplementary Data

Supplementary material 1
