# Supplementary material for: Bovine rhinitis B virus is highly prevalent in acute bovine respiratory disease and causes upper respiratory tract infection in calves
Source: J Gen Virol. 2022 Feb 7;103(2):001714. doi: 10.1099/jgv.0.001714 (PMC8941992; doi:10.1099/jgv.0.001714)
Supplement: Supplementary material 1 [file jgv-103-1714-s001.pdf]

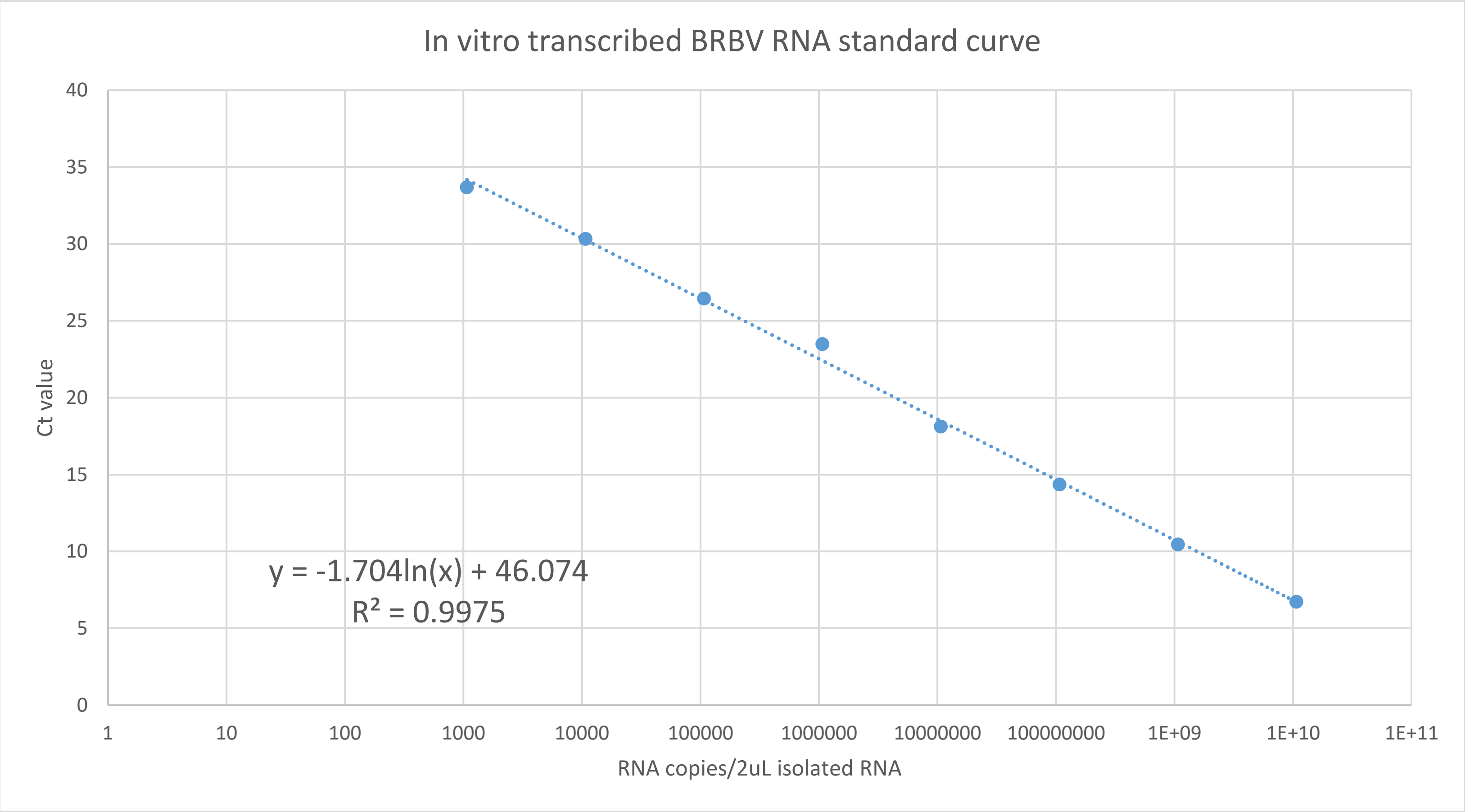

Figure S1. A DNA fragment encompassing the region targeted by the qRT-PCR assay flanked by a 5'-T7 promoter sequence was synthesized and used to generate a synthetic RNA transcript using a MAXIscript kit following manufacturer's instructions. The concentration of the resulting RNA was determined using a Qubit RNA kit following digestion with DNase I.
Serial 10-fold dilutions of synthetic RNA were used to generate a standard curve to correlate RNA copies to qRT-PCR Ct value
